# Ultrathin W space layer-enabled thermal stability enhancement in a perpendicular MgO/CoFeB/W/CoFeB/MgO recording frame

**DOI:** 10.1038/srep16903

**Published:** 2015-11-20

**Authors:** Jae-Hong Kim, Ja-Bin Lee, Gwang-Guk An, Seung-Mo Yang, Woo-Seong Chung, Hae-Soo Park, Jin-Pyo Hong

**Affiliations:** 1 Division of Nano-Scale Semiconductor Engineering, Hanyang University, Seoul 133-791, South Korea; 2 Reserach Institute for Convergence of Basic Science, Novel Functional Materials and Devices Lab, Department of Physics, Hanyang University, Seoul 133-791, South Korea; 3 Nano Quantum Electronics Lab, Department of Electronics and Computer Engineering, Hanyang University, Seoul 133-791, South Korea

## Abstract

Perpendicularly magnetized tunnel junctions (p-MTJs) show promise as reliable candidates for next-generation memory due to their outstanding features. However, several key challenges remain that affect CoFeB/MgO-based p-MTJ performance. One significant issue is the low thermal stability (*Δ*) due to the rapid perpendicular magnetic anisotropy (PMA) degradation during annealing at temperatures greater than 300 °C. Thus, the ability to provide thermally robust PMA characteristics is a key steps towards extending the use of these materials. Here, we examine the influence of a W spacer on double MgO/CoFeB/W/CoFeB/MgO frames as a generic alternative layer to ensure thermally-robust PMAs at temperatures up to 425 °C. The thickness-dependent magnetic features of the W layer were evaluated at various annealing temperatures to confirm the presence of strong ferromagnetic interlayer coupling at an optimized 0.55 nm W spacer thickness. Using this W layer we achieved a higher *Δ* of 78 for an approximately circular 20 nm diameter free layer device.

Spin-transfer-torque magnetoresistive random access memory (STT-MRAM) has emerged as a promising alternative to meet the demand of next-generation non-volatile memories beyond a feature size of 20 nm due to its low power consumption and high-speed performance^1^,^2^,^3^. Recent studies have shown that magnetic tunnel junctions (MTJs) having a perpendicular easy axis (*p*-MTJ) have advantages such as efficient current switching and high thermal stability (*Δ*) as compared to in-plane MTJs (*i*-MTJs)^3^,^4^,^5^.

A variety of perpendicular magnetic anisotropy (PMA) materials have been employed to obtain these features including rare-earth/transition metal alloys and (Co,Fe)/(Pd,Pt) or their L_1_0-ordered alloys^6^,^7^,^8^,^9^. In particular, *p*-MTJs based on CoFeB/MgO frames show promise as reliable building blocks due to their high tunneling magnetoresistance (TMR) ratio^10^. However, these materials still have the disadvantage of a low thermal stability, which can be expressed as *Δ* = E_B_/*k*_*B*_T = *K*_*eff*_V*/k*_*B*_T. Here, E_B_, *K*_*eff*_, *V*, *k*_*B*_, and *T* represent the energy barrier, effective magnetic anisotropy energy density, volume of the magnetic layer, Boltzmann constant, and absolute temperature, respectively. The *Δ* rapidly deteriorates as the size of CoFeB/MgO-based MTJ decreases below a critical diameter. Therefore, achieving higher *Δ* values for specifically reduced dimensions is a challenging issue^11^. Thus, in an attempt to enhance E_B_ (equivalent to *K*_*eff*_V), previous works have sought to enhance *K*_*eff*_ via the introduction of materials with large PMA values^12^,^13^,^14^. In recent years, double CoFeB/MgO frames containing a metal spacer have received great interest as the most prominent way to increase the effective volume factor, V, by increasing the recording thickness^15^,^16^,^17^,^18^. Sato *et al*. introduced a Ta metal spacer-based CoFeB/Ta/CoFeB recording frame that showed increased thermal stability; however, the widespread use of CoFeB/MgO frames with a Ta layer is limited because thermally-activated Ta diffusion during high-temperature annealing results in a degradation of the PMA characteristics^19^. Thus, obtaining an enhanced *Δ* in double CoFeB/MgO frames containing a suitable metal spacer at high annealing temperatures could facilitate the development of STT-MRAM devices.

In this study, we report the effects of W spacers on double MgO/CoFeB/W/CoFeB/MgO frames. These structures exhibited thermal stability even at a high annealing temperature of 425 °C. The interlayer exchange coupling characteristics between two CoFeB layers were systematically examined as a function of the thickness of the W spacer layer. Strong ferromagnetic coupling was achieved at *t*_*W*_ = 0.55 nm along with a higher anisotropic field value, *H*_*K*_, of more than 10 kOe. The extrapolated *Δ* of 78 was increased by a factor of 2.7 without PMA degradation compared with that of a single CoFeB/MgO frame. Therefore, the use of W material in double CoFeB/MgO frames as a suitable metal spacer may enable the development of practical industrial STT-MRAM devices beyond a feature size of 20 nm.

## Results

Various types of stacks were prepared to examine the values *Δ* at different annealing temperatures. Sample A consisted of a substrate/W (5)/CoFeB (1.2)/MgO (2)/W (5) structure while the structure of Sample B was substrate/W (5)/MgO (2)/CoFeB (1.5)/W (5). Finally, Sample C consisted of a substrate/W (5)/MgO (2)/CoFeB (1.5)/W (0.55)/CoFeB (1.2)/MgO (2)/W (5) structure. For convenience, Samples A and B are denoted as single-CoFeB frames with W buffers and capping layers, respectively, and Sample C is denoted as a double-CoFeB frame. Two different stacks containing a Ta or W metal spacer in a double-CoFeB frame were also prepared for comparison. The former consisted of substrate/W (5)/MgO (2)/CoFeB (1.5)/Ta (*t*_*Ta*_)/CoFeB (1.2)/MgO (2)/W (5) structures with various Ta thicknesses, and these are referred to as the Sample C_Ta_ series. The analogous samples with W consisted of substrate/W (5)/MgO (2)/CoFeB (1.5)/W (*t*_*W*_)/CoFeB (1.2)/MgO (2)/W (5) structures with W layers of varying thicknesses, and these were referred to as the Sample C_W_ series.

**Figure 1:**
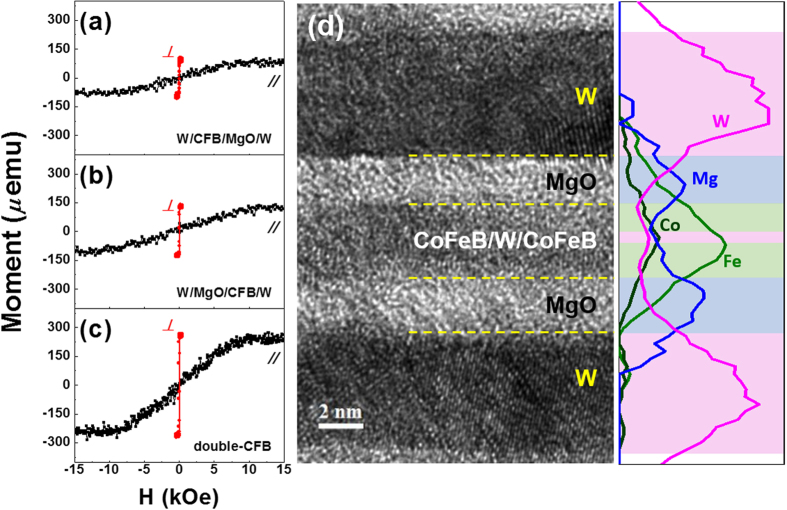
Perpendicularly magnetized W/CoFeB/MgO frames. Typical M-H curves of (**a**) Bottom W (5)/CoFeB (1.2)/MgO (2)/W (5) [Sample A], (**b**) W (5)/MgO (2)/CoFeB (1.5)/Top W (5) [Sample B], and (**c**) W (5)/MgO (2)/CoFeB (1.5)/W (0.55)/CoFeB (1.2)/MgO (2)/W (5) [Sample C]. (**d**) Representative cross-sectional TEM image of Sample C and the corresponding EDS line profile revealing the uniformly well-defined layers in the stacks. All samples were post-annealed at 350 °C and the numbers in parenthesis refer to the nominal layer thickness in nanometers.

Representative in-plane (black line) and out-of-plane (red line) magnetic hysteresis (M-H) loops of Samples A, B, and C annealed at 350 °C are shown in Fig. 1(a–c), respectively. As shown, clear PMA behaviors were observed in all samples. Samples A and B displayed small magnetic moments, while Sample C exhibited a relatively large magnetic moment due to the 2.7 nm thick CoFeB layer.

Sample C was selected as one example from the magnetization curve measurements taken for the Sample C_W_ series. A more detailed W spacer thickness dependence is given in Supplementary Figs S1 and S2, which demonstrate the strong ferromagnetic coupling (FC) behavior in the Sample C_W_ series. FC occurs when the two CoFeB layers act as a single layer in a switching process that will be discussed below. The in-plane saturation field (*H*_*K*_) values of Samples A, B, and C were approximately 10 kOe. This implies that the *K*_*eff*_ was high based on the equation *K*_*eff*_ = *M*_*S*_ ∙ *H*_*K*_/2, where *M*_*S*_ represents the saturation magnetization. The *H*_*K*_ was approximately 10 kOe in all the samples indicating an enhanced *Δ* when a suitable W layer is used in place of a Ta layer. The incorporation of a Ta layer resulted in *H*_*k*_ values of less than 5 kOe in previous work due to the diffusion of thermally-activated Ta atoms from the Ta layer^20^. A representative cross-sectional HR-TEM image of Sample C annealed at 350 °C is displayed in Fig. 4(d) along with the corresponding energy-dispersive X-ray spectroscopy (EDS) line profile. These data reveal uniform, well-defined layers in the stacks. The numbers in parenthesis refer to the nominal layer thickness in nanometers. The W spacer layer is too thin to be observed clearly in this TEM figure. However, the clear peak of W in the middle region of CoFeB/W/CoFeB was detected in the EDS line profile data, which ensures the insertion of an ultrathin W spacer layer.

**Figure 2:**
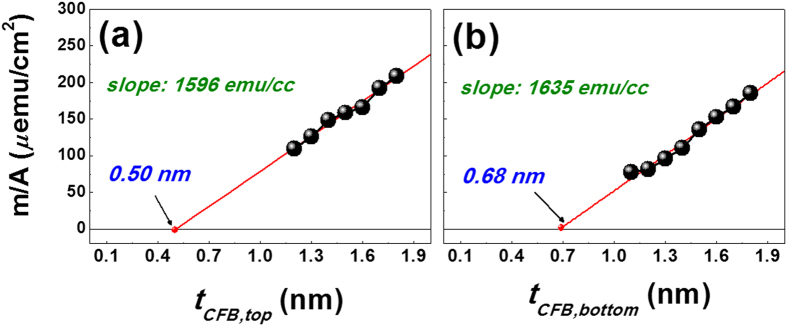
Extrapolated plots of magnetic dead layer. Areal saturation magnetization plots of W (5)/MgO (2)/bottom CoFeB (*t*_*CFB,bottom*_)/W (0.55)/top CoFeB (*t*_*CFB,top*_)/MgO (2)/W (5) stacks as a function of (**a**) top and (**b**) bottom CoFeB thickness recorded after 350 °C annealing. The magnetic dead-layer values (Top: 0.50, Bottom: 0.68 nm) and 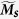 values (Top: 1596, Bottom: 1635 emu/cc) for the top and bottom CoFeB layers were determined via the intercepts and slopes in the curves, respectively.

Figure 2(a,b) present the areal saturation magnetization (m/A) plots used to determine the magnetic dead layer (MDL) and average saturation magnetizations (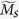

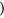
 of the top and bottom CoFeB layers in W (5)/MgO (2)/CoFeB (*t*_*CFB,bottom*_)/W (0.55)/CoFeB (*t*_*CFB,top*_)/MgO (2)/W (5) stacks annealed at 350 °C. The MDLs were utilized to determine the values of *M*_*S*_ = (m/*A)*/t^*^, *K*_*eff*_ = *M*_*S*_ ∙ *H*_*K*_/2, and *Δ* = E_B_*/k*_*B*_T. Here, *M*_*S*_ and t^*^ represent the CoFeB saturation magnetization and the effective thickness of CoFeB (t* = *t*_*CFB*_ − *t*_*d*_), respectively. When the MDLs of the top CoFeB layer were determined, the 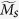 values of the bottom CoFeB layer were excluded and vice versa. As presented in Fig. 2, the MDLs (top: 0.50, bottom: 0.68 nm) and 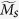 values (Top: 1596, Bottom: 1635 emu/cc) were obtained via the intercepts and slopes in the curves, respectively. The MDL of the bottom CoFeB layer was ~0.2 nm thicker than that of top CoFeB layer. This trend agrees with the results observed for the single CoFeB frames reported by other groups^21^. In addition, our previous work shows that the MDL tends to be thicker in the MgO/CoFeB/W buffer frame than in the W/CoFeB/MgO capping frame^12^. Such a variation in MDL is the result of a separate reaction between the W spacer and CoFeB layer during sputtering^22^.

**Figure 3:**
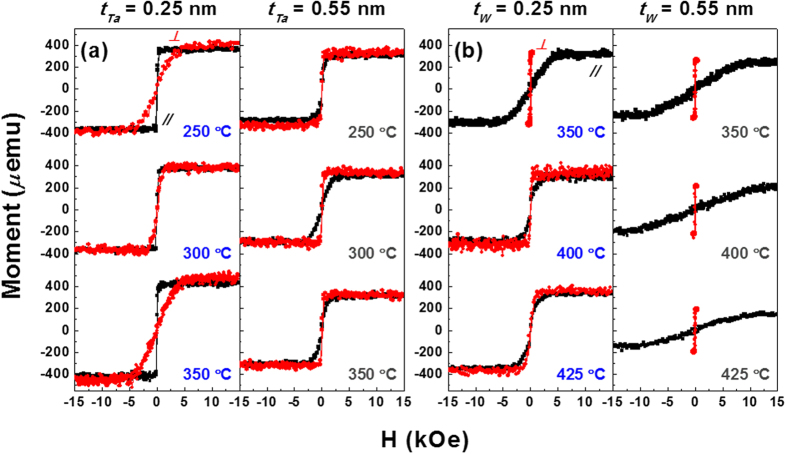
Annealing temperature dependence of perpendicularly magnetized double CoFeB structure. M-H hysteresis responses of W (5)/MgO (2)/CoFeB (1.5)/Ta (*t*_*Ta*_ = 0.25 and 0.55 nm)/CoFeB (1.2)/MgO (2)/W (5) [Left] and W (5)/MgO (2)/CoFeB (1.5)/W (*t*_*W*_ = 0.25 and 0.55 nm)/CoFeB (1.2)/MgO (2)/W (5) [Right] configurations. The Ta-based samples were annealed at 250, 300, and 350 °C, while the W-based samples were annealed at 350, 400, and 425 °C. The 0.55 nm thick W-based sample retained stable PMA features even at 425 °C without significant degradation, reflecting an enhanced thermal stability via replacement of Ta with a proper W space layer.

To further validate the annealing temperature dependence of the PMA, samples from the C_Ta_ series and C_W_ series were systematically annealed at various temperatures. Initially, relatively thin (0.25 nm) and thick (0.55 nm) Ta and W layers were chosen for the PMA measurements for comparison. Furthermore, since the Ta/CoFeB/MgO or MgO/CoFeB/Ta frames typically exhibited degraded PMA responses after high-temperature annealing^19^, Sample C_Ta_ was annealed at relatively low temperatures. For example, Sample C_Ta_ structures with 0.25 and 0.55 nm Ta spacers were annealed at 250, 300, and 350 °C, while Sample C_W_ structures with the same spacer thicknesses were annealed at 350, 400, and 425 °C. As is evident in Fig. 3(a), Sample C_Ta_ with a 0.25 nm Ta thickness showed dominant in-plane magnetic anisotropy (IMA) features over all annealing temperatures. In contrast, Sample C_W_ with *t*_*W*_ = 0.25 nm exhibited a clear PMA feature up to T_a_ = 350 °C with degradation beginning at T_a_ = 400 °C, as shown in Fig. 3(b). It is widely believed that the degraded PMA features in Samples C_Ta_ or C_W_ with ultrathin 0.25 nm spacers arise primarily from out-diffusion of thermally-activated Ta or W atoms towards the CoFeB/MgO interface or inside MgO layer, respectively^12^,^19^, after annealing at temperatures above 300 °C to 400 °C. These diffusion processes affect the interfacial anisotropy (IA) caused by the hybridization of Co and Fe 3*d* orbitals and the O 2*p* orbital. Closer analysis verified the enhancement in PMA characteristics of Sample C_W_ at relatively higher annealing temperatures under the same spacer thickness. For example, Sample C_Ta_ with a 0.55 nm spacer exhibited weak PMA characteristics at 300 °C, while Sample C_W_ with *t*_*W*_ = 0.55 nm revealed clear PMA features up to T_a_ = 425 °C via the replacement of Ta with a proper W layer.

Our previous work^12^ demonstrated that the Ta buffer layer exhibited a nearly amorphous structure, while the W buffer layer revealed a single crystal-like structure. Therefore, the use of the W layer provided annealing stability up to 425 °C. Similarly, the W/CoFeB interface in this work may lead to unique suppression of an inter-diffusion event during annealing, which enhances the *K*_*eff*_ value. As given in Figs S1 and S2, further study of spacer thickness dependence confirmed the presence of a required minimum thickness (*t*_*W*_ = 0.25 nm) for sustaining a perpendicular easy axis during annealing at temperatures higher than 400 °C.

We proposed a simple hypothesis to clarify the above observations regarding the effect of a W spacer. It is well-known that deterioration of PMA features for CoFeB/MgO stacks involving the incorporation of a Ta layer is directly linked to degradation of the IA due to thermally-activated Ta layer atoms diffusing into the CoFeB/MgO interface at a high annealing temperature. Furthermore, the PMA properties at CoFeB/MgO interfaces are strongly affected by various buffer or capping metal layers^12^,^13^,^23^. Thus, the enhancement in the PMA obtained in our work is likely to arise from B affinity. B affinity is an important factor for creating PMA features since boron (B) out-diffusion leads to the formation of well-aligned crystalline structures during annealing^24^. Therefore, promising PMA properties that result from high temperature annealing may arise from effective absorption of the proper amount of B provided by the W spacer. In addition, first principles density-functional calculations have showed that a nonmagnetic metal (NM)/CoFeB interface partially contributed to the PMA^25^. Thus, the W/CoFeB interface may inherently have a higher *H*_*k*_ since certain metal materials contain unoccupied majority-spin d states that could generate an additional perpendicular IA^26^. Finally, the slight cohesive energy difference between Ta (8.1 eV/atom) and W (8.9 eV/atom) is also likely to affect the annealing stability. Suppression of an inter-diffusion event typically caused by high annealing temperatures may occur at the W/CoFeB interface. However, more detailed work is required to clarify the reason for the highly-promising PMA and *Δ* enhancement observed for the W spacer in the Sample C_W_ series.

**Figure 4:**
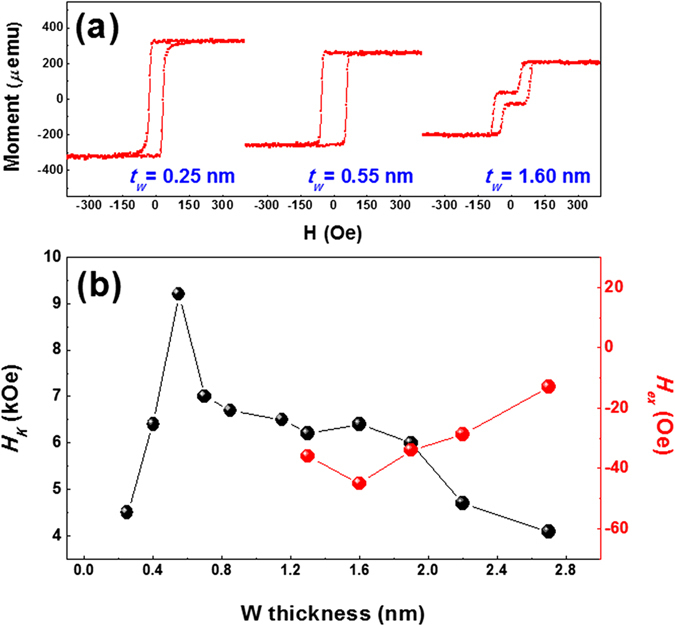
Exchange coupling behaviors as a function of W thickness. (**a**) Perpendicular M-H hysteresis curves of W (5)/MgO (2)/CoFeB (1.5)/W (*t*_*W*_)/CoFeB (1.2)/MgO (2)/W (5) stacks as a function of W space layer thickness. Variation in the W thickness represents a transition from ferromagnetic to anti-ferromagnetic coupling behaviors at a specific W thickness. (**b**) Dependence of in-plane saturation field (black dots, *H*_*k*_) and field shift of minor loops (red dots, *H*_*ex*_) on the W thickness.

To gain insight into how the W spacer thickness influences the exchange coupling behavior and anisotropic field *H*_*K*_, M-H loops of a Sample C_W_ series specimen annealed at 350 °C were recorded. These measurements were taken in the presence of a small applied field in the range of ±0.5 kOe, and *t*_*W*_ was varied from 0.10 to 2.70 nm. Figure 4(a) shows the representative M-H hysteresis loops of Sample C_W_ with spacer thicknesses of 0.25, 0.55, and 1.60 nm. As is evident in this figure, strong FCs were obtained for specimens with *t*_*W*_ = 0.25 and 0.55 nm. In addition, the magnetic moments decreased with increasing W thickness, which is in agreement with the results for Ta thickness dependence reported in our previous work^20^. The sample with *t*_*W*_ = 1.60 nm exhibited clear anti-ferromagnetic coupling (AFC) behavior. As seen in Fig. S1, a transition from FC to AFC behavior was observed above *t*_*W*_ = 1.15 nm.

To determine the optimum W thickness for higher thermal stability, the change in anisotropic field *H*_*K*_ (black circle) and field shift *H*_*ex*_ (red circle) were plotted as a function of *t*_*W*_ in Fig. 4(b). Based on the formula for thermal stability, *Δ* = E_B_*/k*_*B*_T, a larger energy barrier (E_B_) is required for higher *Δ*, if the *K*_*eff*_ value remains the same. As *t*_*W*_ increases, *H*_*K*_ reaches its peak value of approximately ~10 kOe at *t*_*W*_ = 0.55 nm. This point represents the highest *K*_*eff*_ value of approximately ~6 Merg/cc, and it then starts to decrease.

A similar pattern was observed at higher annealing temperatures, as shown in Figs S1 and S2. The highest *K*_*eff*_ value at 400 °C was approximately ~7 Merg/cc for the sample with *t*_*W*_ = 0.55 nm. To estimate the exchange coupling strength based on the equation *J* = *H*_*ex*_*M*_*S*_ *t*, the field shift, *H*_*ex*_, was measured for various *t*_*W*_ values, as seen in Fig. 4(b). The coupling strength monotonically decreased up to *t*_*W*_ = 1.60 nm and then began to increase. It is well-known that the use of several NM interlayers for ferromagnetic metal (FM)/NM/FM frames allows for the wide-range FC behavior until the thickness meets the criteria for an AFC transition^27^. The W interlayer used in this work showed similar behavior. As the W thickness increased, the ferromagnetic coupling strength weakened, and oscillatory behavior occurred due to RKKY- (*Ruderman-Kittel-Kasuya-Yosida*) type coupling; however, a second oscillation peak was not observed in our frames^27^. Coupling behavior was ambiguous at higher annealing temperatures. Therefore, it is not clear if RKKY-type coupling is dominant in our frames. As such, more experiments are needed to establish a specific explanation for the origin of the interlayer exchange coupling observed in our frames.

**Figure 5:**
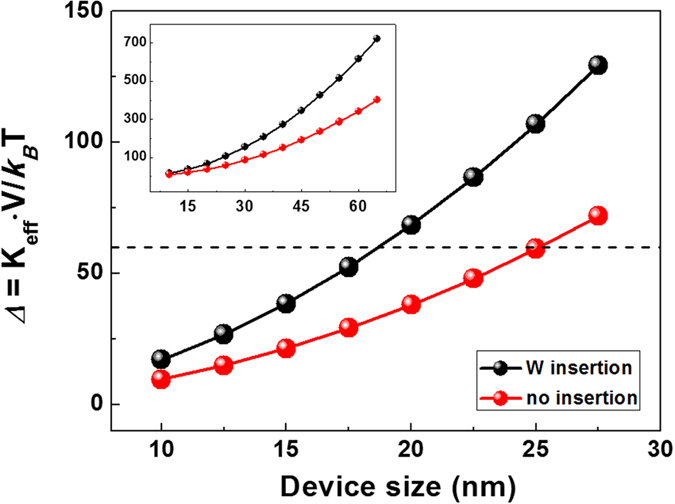
Calculated thermal stability factor. Thermal stability factor (*Δ* = *K*_*eff*_*V*/*k*_*B*_*T*) plots for W (5)/MgO (2)/CoFeB (1.5)/W (0.55)/CoFeB (1.2)/MgO (2)/W (5) [black dots] and W (5)/MgO (2)/CoFeB (1.5)/W (5) [red dots] frames versus circular-shaped device size. The volumes of the CoFeB dead layer and W space layer were not included in the *Δ* calculation. All samples were annealed at 400 °C. Sample C, which contained a W space layer with a surface diameter of 20 nm, revealed a 2.7-fold enhancement in thermal stability over that of a single CoFeB frame without a W space layer. The inset shows a plot of the *Δ* factors in a large-scale device.

To further confirm the observed *Δ* behaviors, the *Δ* was calculated as a function of circular device size for both W (5)/MgO (2)/CoFeB (1.5)/W (0.55)/CoFeB (1.2)/MgO (2)/W (5) and W (5)/MgO (2)/CoFeB (1.5)/W (5) frames annealed at *T*_*a*_ = 400 °C, as shown in Fig. 5. Previous investigators reported that the (effective) anisotropy energy *K*_*U*_ (*K*_*eff*_) does not decrease as the device size shrinks^28^,^29^. Therefore, the equation frequently used for the *Δ* calculation in larger devices was also employed to estimate the performance of nano-scaled devices suitable for real MRAM applications^28^. The circular device volume was determined according to the relation V=A∙ *t*_CFB_*. Here, we employed the effective CoFeB magnetic layer thickness and we excluded the magnetic dead layer (more details related to the magnetic dead layers of samples annealed at *T*_*a*_ = 400 °C are given in Fig. S3 of the Supporting Information).

## Discussion

The *Δ* of Sample C_W_ was approximately 78 for the 20 nm diameter device, which satisfies the well-known criterion for key demands. This value was a factor of 2.7 larger than that observed in the single CoFeB frame. Therefore, this material is a promising alternative for use in the development of STT-MRAM. In addition, other reports have been published on the successful reduction in *J*_*C*_ caused by an inserted spacer^30^, even though the influence of the W spacer on the *J*_*C*_ was not addressed in this work. The observed *M*_*S*_ of Sample C_W_ was ~1250 emu/cc, which was comparable to the value obtained from the single CoFeB frame. The similarity in the *M*_*S*_ values implies that *J*_*C*_ is likely to be similar even upon incorporation of a W spacer. However, since the origin of enhanced thermal stability in the Sample C_W_ series has not been clarified, more study is necessary to verify the possible role of the W spacer.

In this work, we reported the thermal stability of double-CoFeB frames with a W spacer at temperatures up to 425 ^o^C, which meet the demand of the BEOL process. Strong ferromagnetic coupling was observed with a 0.55 nm thick W layer along with a *H*_*K*_ value of approximately ~10 kOe. These results suggest that we achieved enhanced thermal stability in double-CoFeB frames. The *Δ* of a 20 nm device increased by a factor of 2.7 compared to that of a single CoFeB free layer. Thus, we anticipate that the ability to improve thermal stability via the use of W spacers will be useful in practical applications even though the origin of the improved thermal stability is not specifically known.

## Methods

All samples were deposited onto thermally-oxidized Si wafers at room temperature via dual DC and RF magnetron sputtering (SciEN Tech system) with a Co_20_Fe_60_B_20_ target. The numbers in parenthesis refer to the layer thickness in nanometers. The deposition was carried out at a working pressure of 3 × 10^−3^ Torr with a base pressure of less than 5 × 10^−8^ Torr. For the Sample C_W_ series, the W thickness was varied from 0.10 to 2.70 nm, in which the top (bottom) CoFeB thickness was also varied within the range of 1.2 (1.1) to 1.8 nm to determine the magnetic dead layers of the top (bottom) CoFeB layers. The samples were annealed at various temperatures ranging from 250 to 425 °C for 1 hour under vacuum pressures below ~1 × 10^−6^ Torr with a 30 kOe magnetic field applied normal to the sample plane. A vibrating sample magnetometer (VSM) was utilized to analyze the in-plane and out-of-plane magnetic hysteresis loops, along with a structural investigation performed via high-resolution transmission electron microscopy (HR-TEM).

## Additional Information

**How to cite this article**: Kim, J.-H. *et al*. Ultrathin W space layer-enabled thermal stability enhancement in a perpendicular MgO/CoFeB/W/CoFeB/MgO recording frame. *Sci. Rep*. **5**, 16903; doi: 10.1038/srep16903 (2015).

## Supplementary Material

Supplementary Information
