# Supplementary material for: Ultrathin W space layer-enabled thermal stability enhancement in a perpendicular MgO/CoFeB/W/CoFeB/MgO recording frame
Source: Sci Rep. 2015 Nov 20;5:16903. doi: 10.1038/srep16903 (PMC4653616; doi:10.1038/srep16903)
Supplement: Supplementary Information [file srep16903-s1.pdf]

Supplementary Figures of

Ultrathin W space layer-enabled thermal stability enhancement in a perpendicular MgO/CoFeB/W/CoFeB/MgO recording frame

Jae-Hong Kim[1], Ja-Bin Lee[2], Gwang-Guk An[2], Seung-Mo Yang[2], Woo-Seong Chung[3], Hae-Soo Park[2] & Jin-Pyo Hong[1,2,*]

[1]Division of Nano-Scale Semiconductor Engineering, Hanyang University, Seoul 133-791, South Korea,

[2]Research Institute for Natural Science, Novel Functional Materials and Devices Lab, Department of Physics, Hanyang University, Seoul 133-791, South Korea

[3]Nano Quantum Electronics Lab, Department of Electronics and Computer Engineering, Hanyang University, Seoul 133-791, South Korea,

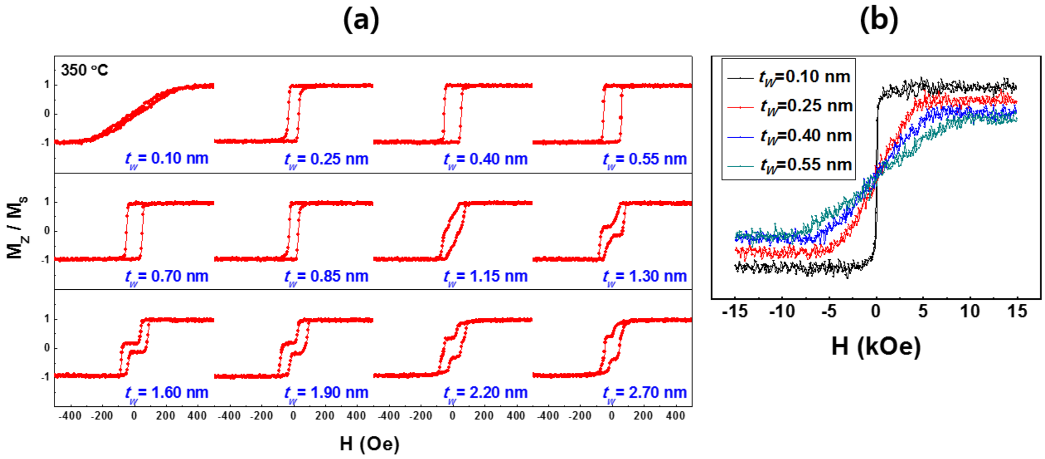

Figure S1. (a) Out-of-plane hysteresis behaviors of 350 °C-annealed W (5)/MgO (2)/CoFeB (1.5)/W (t_W)/CoFeB (1.2 nm)/MgO (2 nm)/W (5 nm) stacks as a function of W thickness, in which W thickness was varied from 0.10 to 2.70 nm. Variation in W thickness revealed the presence of strong ferromagnetic coupling (FC) at t_W=0.55 nm and then the transition from FC to antiferromagnetic coupling (AFC) behaviors. Further increasing W thickness resulted in gradually weakening AFC strength. (b) Representative in-plane hysteresis curves of samples observed in the first top row of Fig. S1(a), exhibiting the H_k peak at t_W=0.55.

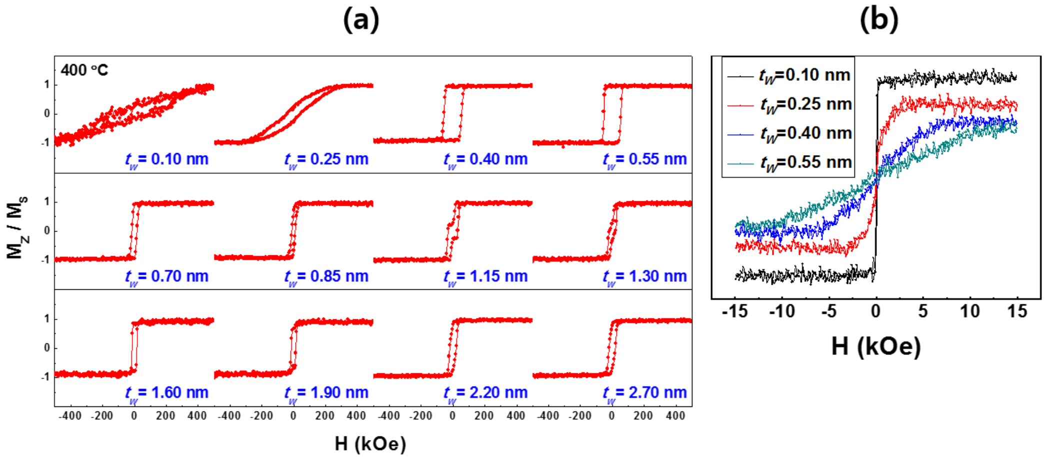

Figure S2. (a) Out-of-plane hysteresis loops of 400 ^{o}C-annealed W (5)/MgO (2)/CoFeB (1.5)/W (t_W)/CoFeB (1.2)/MgO (2)/W (5) stacks as a function of W thickness. A strong FC was observed at t_W=0.55 nm; however, AFC features easily disappeared at higher annealing temperatures. (b) Representative in-plane hysteresis curves of samples observed in the first top row of Fig. S1 (a), exhibiting the H_k peak at t_W=0.55.

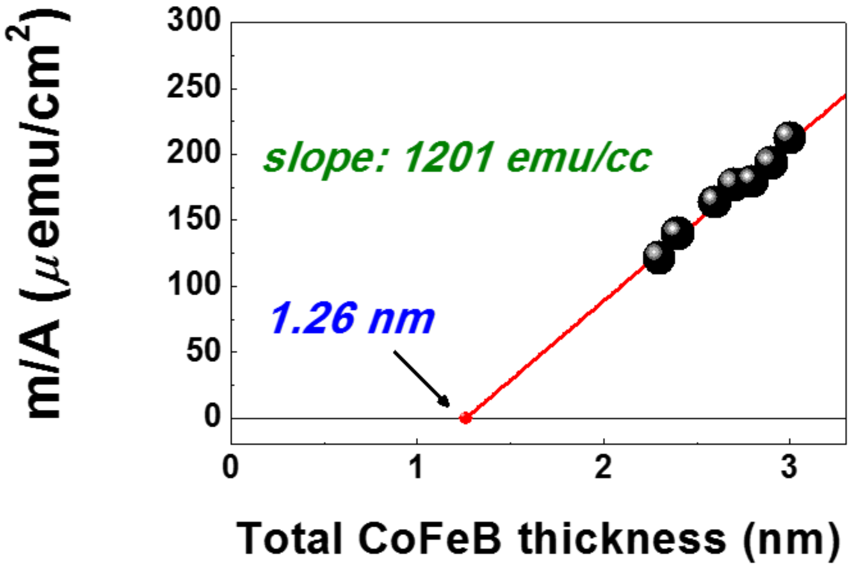

Figure S3. Areal saturation magnetizations (M/A) plots of W (5)/MgO (2)/ bottom CoFeB ($t_{CFB,bottom}$)/W (0.55)/top CoFeB ($t_{CFB,top}$)/MgO (2)/W (5) stacks as a function of total CoFeB thickness consisting of top and bottom CoFeB thickness. The samples were annealed at 400 ℃. The dead layer (1.26 nm) thickness determined in this curve was used to evaluate thermal stability factors depicted in Figure 5. A higher annealing temperature (in this case, 400 ℃) resulted in a reduced $\widetilde{M_s}$, when compared with that annealed at 350 ℃.
